# Clustered ERF Transcription Factors: Not All Created Equal

**DOI:** 10.1093/pcp/pcaa067

**Published:** 2020-05-11

**Authors:** Ling Yuan

**Affiliations:** p1 Department of Plant and Soil Sciences, Kentucky Tobacco Research and Development Center, University of Kentucky, Lexington, KY 40546, USA; p2 South China Botanical Garden, Guangzhou, China

**Keywords:** Alkaloid biosynthesis, ERF transcription factors, Gene clusters, Nicotine, Transcriptional regulation

Plants synthesize >200,000 natural products, also known as specialized metabolites or secondary metabolites. Acting as defense compounds, pigments or hormonal signal molecules, specialized metabolites enable plants to interact with their biotic and abiotic environments. While plants produce the metabolites for their own growth, defense and stress tolerance, humans have a long history of exploiting plant natural products to promote health and to treat diseases. Taxol, a terpenoid from *Taxus brevifolia*, and vinblastine and vincristine, terpenoid indole alkaloids from *Catharanthus roseus*, are clinical anticancer drugs. Other natural products are well known for their antioxidant and anti-inflammatory activities, many of which are alkaloids, e.g. theobromine and paraxanthine from cocoa, caffeine and gingerol and shogaols from ginger.

Plant alkaloids are one of the largest and most fascinating families of specialized metabolites. Alkaloids are nitrogen-containing molecules with the number of known structures exceeding 20,000. Some alkaloids have neuroactivities, such as morphine, caffeine and nicotine. Nicotine is also a highly effective insecticide, binding to the nicotinic acetylcholine receptors of an insect’s central nervous system. Some tobacco varieties accumulate nicotine to up to 3% of the leaf dry weight. One benefit is that tobacco crops commonly experience significantly less herbivore damages than other agricultural crops. Nicotine is synthesized in tobacco roots and transported upward from roots to aerial tissues through the xylem (Fig. 1). Most of the newly synthesized nicotine is sequestrated in the vacuoles of root cells. When tobacco leaves are damaged by insect attack or mechanical wounding, nicotine biosynthesis spikes, triggered by the phytohormone jasmonate (JA), which moves downward from leaves to roots. From a molecular biology perspective, the tissue-specific biosynthesis, quick response to JA induction and rapid transport and uploading of nicotine make tobacco an excellent model for studying gene regulation of alkaloid biosynthesis.

**Fig. 1:**
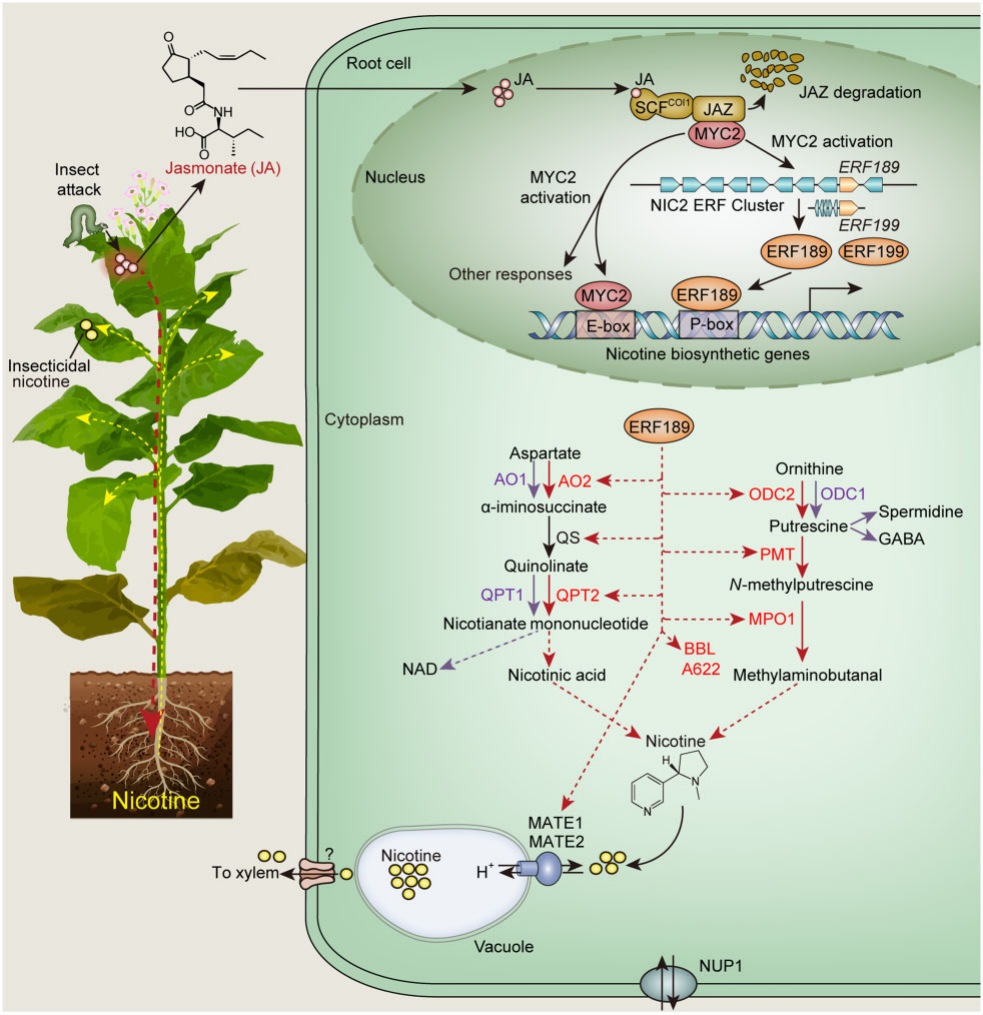
The role of the NIC2 ERF cluster in the activation of the nicotine biosynthetic pathway. Attack by insects activates the production of JA, which moves downward to the root. JA is transported into root cells and subsequently converted to methyl-JA, which induces SCF^COI1^-mediated degradation of Jasmonate ZIM-domain proteins (JAZ). Upon JAZ degradation, the master regulator MYC2, sequestered by JAZ in the absence of JA, is freed to activate the ERF cluster genes. MYC2, in addition to activating NIC2 cluster genes, coactivates the nicotine pathway genes with ERF189. Not all NIC2 members are significantly involved in the activation of the nicotine biosynthetic pathway, which starts from aspartate and ornithine. ERF189 (likely also ERF199) selectively activates the enzymes of the nicotine pathway (indicated by red dash arrows), which overlaps with several primary metabolic pathways (i.e. NAD, spermidine and GABA, indicated by purple arrows). Newly synthesized nicotine is transported into vacuoles by transporters, MATE1 and MATE2, which are also regulated by ERF189. NUP1 and other unidentified transporters are involved in the nicotine transport. Insecticidal nicotine, especially under the induction of JA, is transported out of the vacuoles, moved upward to vegetative tissues through the xylem and uploaded into leaf cells by NUP1. AO, aspartate oxidase; QS, quinolinate synthase; quinolinate phosphoribosyltransferase; ODC, ornithine decarboxylase; PMT, putrescine *N*-methyltransferase; MPO, *N*-methylputrescine oxidase; BBL, berberine bridge enzyme-like proteins; A622, PIP-family protein; NUP, purine permease transporter.

Understanding the gene regulation of nicotine biosynthesis is of practical and basic scientific interests. The ability to produce tobacco with high- or low-nicotine content is by no means trivial. High-nicotine tobacco can be used in nicotine extraction for medicinal purposes. Low-nicotine tobacco leaves are potentially useful in combating nicotine addiction. Nicotine contains a pyridine ring and a pyrrolidine ring, derived from aspartate and ornithine, respectively, in a multienzyme process (Fig. 1). Nicotine biosynthesis competes with the NAD and polyamine primary metabolic pathways, thus requiring the control of a special set of gene regulators. Like other specialized metabolites, nicotine biosynthesis is mainly controlled at the transcriptional level (Colinas and Goossens 2018). The nicotine biosynthetic genes are predominantly regulated by two clusters of JA-responsive Apetala 2/ethylene response factor (ERF) transcription factors (TFs), which originated from the two tobacco progenitors, *Nicotiana tomentosiformis* and *Nicotiana sylvestris*. In addition to the tobacco ERF clusters (Kajikawa et al. 2017), recent years have seen the characterization of JA-responsive ERF clusters in potato and tomato (Cardenas et al. 2016, Thagun et al. 2016), *C. roseus* (Paul et al. 2017) and other plants (Singh et al. 2020). In contrast to gene clusters encoding nonhomologous metabolic enzymes (Nutzmann et al. 2016), the ERF clusters consist of homologous genes in the tandem organization (Kajikawa et al. 2017, Singh et al. 2020). Repeated gene duplication, mainly through unequal crossing-over events, likely contributes to the formation of such clusters (Magadum et al. 2013). Phylogenetic relationships of clustered *ERF*s from various plant families suggest independent, multiple emergences of the clusters in different lineages.

The nature of evolution by gene duplication begs the question of whether the members of ERF clusters are merely duplicate copies of one another with redundant function. Recent advances in understanding ERF clusters point to the contrary. First, intra-cluster regulatory relationships have been demonstrated in the *C. roseus* ORCA cluster and tobacco NIC2 cluster (Paul et al. 2020). Second, Shoji et al. (2010) have shown that ERF189, of the NIC2 cluster from *N. tomentosiformis*, and its closest homolog ERF199, from *N. sylvestris*, play predominant regulatory roles in nicotine biosynthesis, while other members in the NIC2 cluster play only minor or no roles. The loss of ERF189 function dramatically reduces alkaloid production (Shoji et al. 2010). Expression profiles of ERF genes also support the differential regulation of nicotine biosynthesis by ERF189/ERF199. In contrast to the low-level expression of other NIC2 ERF genes, *ERF189/ERF199* seem to be highly expressed in roots where nicotine is synthesized (Kajikawa et al. 2017). Recent work in tomato shows that, within an ERF cluster, the *GAME9* (*JRE4*) gene predominantly regulates steroidal glycoalkaloid biosynthesis (Cardenas et al. 2016, Nakayasu et al. 2018), further strengthening the notion that individual ERFs in a cluster possess unique functions. From a bioengineering perspective, the unique functions of individual ERFs suggest that knocking out a single gene in a cluster (not the entire cluster) will significantly alter the metabolic outcomes.

Reported in this issue of *Plant and Cell Physiology*, Hayashi et al. (2020) tested the ability to alter nicotine production by controlling the expression of *ERF189* and *ERF199* and determined the metabolic outcomes of the genetic manipulation. First, the authors confirmed the root-specific expression of *ERF189*/*ERF199* and their orthologs in two other *Nicotiana* species. Next, they overexpressed *ERF189*, alone or with *MYC2* (see Fig. 1), and measured the effects on nicotine-related gene expression and alkaloid accumulation. *ERF189* overexpression was achieved in multiple ways, including transient expression in leaves of *Nicotiana benthamiana* and *Nicotiana alata* (normally containing no nicotine in leaves), as well as stable transgenic tobacco plants overexpressing *ERF189*, controlled by either the constitutive *CaMV35S* promoter or the leaf-specific tomato *rbcS* promoter. This approach is insightful and allows the evaluation of the effects of ERF189 in the leaf without the interference of root-derived nicotine (in the case of *N. alata* transient expression and *rbsS*-driven overexpression). Overexpression of *ERF189* alone, or in combination with *MYC2*, significantly induced the expression of nicotine pathway genes (more so in the *ERF189*-*MYC2* combination) and consequently increased alkaloid accumulation in leaves, but not in roots. The authors also created tobacco *erf189/erf199* double mutants by CRISPR/Cas9-mediated genome editing. In the complete knockout mutants, alkaloid levels are <4% of the control in leaves, although higher in roots, indicating the presence of additional root-specific regulators. Interestingly, in one of the mutants in which *ERF199* is knocked out while *ERF189* contains a substitution mutation that presumably only reduces its activity, the alkaloid level was approximately 50% to that of the control. This work demonstrates that the nicotine level can be dialed up and down by manipulating one or two ERFs.

The work by Hayashi et al. (2020) also provides insight into the metabolic impacts of genetically manipulating the ERF genes. The authors profiled the metabolic outcomes in leaves overexpressing *ERF189* and in roots of the ERF double knockout mutant. *ERF189* overexpression resulted in increased nitrogen-containing metabolites, which were decreased in the knockout plants. The knockout plants are phenotypically normal; however, the metabolites are significantly altered in the roots, suggesting that the plant can compensate the genetic reprogramming.

This work is elegant and informative. It further affirms that ERF189/ERF199 in the ERF clusters are the primary regulators of nicotine biosynthesis. While it raises additional questions, some existing questions are still left unanswered. For instance, what then are the functions of other homologs in the cluster? They may participate in the regulation of *ERF189* (Paul et al. 2020) or in other unrelated processes as some of them are strongly induced in response to salt stress (Kajikawa et al. 2017). In addition, overexpression of *ERF189* apparently stunts growth, in contrast to heterologous overexpression of its ortholog, *GAME9* (*JRE4*), in tobacco (Nakayasu et al. 2018). Follow-up questions would thus concern the global impacts of ERF189 on both primary and secondary metabolism. Nonetheless, our understanding of TF clusters is still insufficient. Future investigations will include the evolution of TF clusters and the position of a cluster in the global gene regulatory network. How do signal transduction cascades respond to developmental and environmental cues to differentially regulate the members of a TF cluster? Furthermore, by comparison, ERF clusters have been studied more extensively than others, e.g. those formed by MYB or bHLH TFs. Do the evolutionary and functional characteristics of ERF clusters, such as distinct functions of individual TFs in a cluster and intra-cluster regulation, also hold true for other TF clusters? The current explosion of whole-genome sequencing information will no doubt accelerate the discovery of new TF clusters. The results from this publication and other related recent advancements have put spotlights on the gene regulatory hubs consisting of TF clusters.

## Disclosures

The authors have no conflicts of interest to declare.
